# Antenatal multiple micronutrient supplementation: call to action for change in recommendation

**DOI:** 10.1111/nyas.14271

**Published:** 2019-11-05

**Authors:** Megan W. Bourassa, Saskia J.M. Osendarp, Seth Adu-Afarwuah, Saima Ahmed, Clayton Ajello, Gilles Bergeron, Robert Black, Parul Christian, Simon Cousens, Saskia de Pee, Kathryn G. Dewey, Shams El Arifeen, Reina Engle-Stone, Alison Fleet, Alison D. Gernand, John Hoddinott, Rolf Klemm, Klaus Kraemer, Roland Kupka, Erin McLean, Sophie E. Moore, Lynnette M. Neufeld, Lars-Åke Persson, Kathleen M. Rasmussen, Anuraj H. Shankar, Emily Smith, Christopher R. Sudfeld, Emorn Udomkesmalee, Stephen A. Vosti

**Affiliations:** 1 The New York Academy of Sciences, New York, New York; 2 Micronutrient Forum, Washington, D.C; 3 Division of Human Nutrition and Health, Wageningen University, Wageningen, the Netherlands; 4 Department of Nutrition and Food Science, University of Ghana, Legon, Accra, Ghana; 5 The Vitamin Angels Alliance, Inc., Santa Barbara, California; 6 Johns Hopkins Bloomberg School of Public Health, Baltimore, Maryland; 7 Bill & Melinda Gates Foundation, Seattle, Washington; 8 London School of Hygiene and Tropical Medicine, London, United Kingdom; 9 UN World Food Programme, Rome, Italy; 10 Friedman School of Nutrition Science and Policy, Tufts University, Boston, Massachusetts; 11 Department of Agricultural and Resource Economics, University of California, Davis, Davis, California; 12 International Centre for Diarrhoeal Disease Research, Dhaka, Bangladesh; 13 UNICEF, Copenhagen, Denmark; 14 The Pennsylvania State University, University Park, Pennsylvania; 15 Division of Nutritional Sciences, Cornell University, Ithaca, New York; 16 Helen Keller International, Baltimore, Maryland; 17 Sight and Life Foundation, Basel, Switzerland; 18 UNICEF, New York, New York; 19 Department of Women and Children’s Health, King’s College London, London, United Kingdom; 20 Global Alliance for Improved Nutrition GAIN, Geneva, Switzerland; 21 London School of Hygiene and Tropical Medicine, Addis Ababa, Ethiopia; 22 Department of Global Health and Population, Harvard T.H. Chan School of Public Health, Boston, Massachusetts; 23 Summit Institute of Development, Mataram, Indonesia; 24 Institute of Nutrition, Mahidol University, Nakhon Pathom, Thailand; 25 Nuffield Department of Medicine, Centre for Tropical Medicine and Global Health, University of Oxford, Oxford, United Kingdom; 26 Departments of Global Health, and Exercise and Nutrition Sciences, Milken Institute School of Public Health, The George Washington University, Washington, D.C

We appreciate the comments by Devakumar *et al*.^1^ and agree that there are still some unanswered questions regarding the long-term impact of multiple micronutrient supplementation (MMS) during pregnancy. However, in their assessment, Devakumar and colleagues ignore the significant benefits shown in the individual patient data (IPD) meta-analysis, which strongly influenced our task force’s conclusions. Rather, their comments focus only on the birth size data from the *Cochrane* reviews.^2,3^ In the IPD meta-analysis, which included data from nearly 113,000 pregnancies, the authors found that, in addition to reducing the risk of low birthweight, MMS significantly reduces the risk of preterm birth (RR = 0.93 (0.87–0.98), random effects).^2^ The *Cochrane* review also states that MMS “probably led to as light reduction in preterm births” on the basis of data from 91,425 participants with moderate quality evidence (RR = 0.95 (0.90–1.01)).^3^

One of the greatest strengths of an IPD meta-analysis is the ability to include predefined subgroup analyses that are based on the individual data, rather than trial-level aggregate data. The meta-analysis in the *Cochrane* review only used the latter. In the IPD meta-analysis, the authors found that MMS (compared with iron and folic acid supplementation) significantly reduced neonatal mortality among female infants (RR = 0.85 (0.75–0.96)), and had similar significant effects on 6-month and infant mortality, with no effect among males, and reduced 6-month mortality (RR = 0.71 (0.60–0.86)) among all infants born to anemic mothers.^2^ The subgroup analyses also showed that the benefit of MMS with regard to reducing the risk of preterm birth (RR = 0.84 (0.78–0.91)) was greater among infants born to under-weight mothers than among those born to women who were not underweight. The global prevalence estimates of underweight (>200 million women of reproductive age)^4^ and anemia (>528 million women of reproductive age)^5^ are very high, and prevalence rates are highest in low- and middle-income countries, where these MMS trials took place. Thus, in such settings, MMS is likely to benefit a substantial proportion of infants. The potential benefits of MMS for women who are depleted in nutrients other than iron and folate also merit further exploration.

In addition to the short-term benefits of MMS with regard to birth outcomes, the likely longer term consequences of reducing low birthweight and preterm birth should be considered. Low birthweight infants are at an increased risk of death not just during infancy but throughout life, and low birthweight is also associated with reduced lung capacity and immune function as well as an increase in certain cancers.^6^ Reducing low birthweight rates remains a World Health Assembly target, and interventions that can reduce the risk of low birthweight, such as MMS, should be encouraged.

More information would be useful regarding the long-term benefits or consequences of all prenatal interventions. For MMS, the evidence continues to accrue from long-term follow-up of trial participants. We note that the Devakumar *et al*. standard aggregated data meta-analysis includes mortality and anthropometric data for follow-up periods ranging from 6 months to 9 years after birth and combines studies that did and did not continue nutritional interventions after birth, making direct comparisons across studies difficult.^7^ Moreover, there is a risk of bias in the existing evidence from follow-up studies because of loss to follow up due to mortality. Valid and reliable cognitive assessments in very young children remain a challenge, which reduces the likelihood of detecting intervention group differences; a much wider array of assessments can be conducted among older children and adults. Recent results on adolescent cognition from a study in rural Western China are encouraging (and appear to have been overlooked by Devakumar *et al*. in their letter).^8^ In that study, which included more than 2,100 14-year olds, the investigators found that children whose mothers received MMS during pregnancy had a dose-dependent improvement in intellectual development. The findings of that study are similar to those of the follow-up study of school-age children in the Indonesian SUMMIT study at 9–12 years of age.^9^

On the basis of the evidence provided by the *Cochrane* review and the IPD meta-analysis, the task force is of the opinion that there is sufficient evidence to inform policy decisions now, without waiting for more evidence on long-term outcomes. We agree with Devakumar *et al*. that improving health outcomes through nutrition interventions requires a life-course approach. MMS is just one intervention during one phase of the life course, and pregnant women in food-insecure situations may need improved macro- as well as micronutrient intake. That said, in populations at risk, MMS during pregnancy can allow infants to begin life with a significant advantage.
